# Non-Invasive Quantification of White and Brown Adipose Tissues and Liver Fat Content by Computed Tomography in Mice

**DOI:** 10.1371/journal.pone.0037026

**Published:** 2012-05-16

**Authors:** Marko Lubura, Deike Hesse, Nancy Neumann, Stephan Scherneck, Petra Wiedmer, Annette Schürmann

**Affiliations:** Department of Experimental Nutrition, German Institute of Human Nutrition Potsdam-Rehbruecke, Nuthetal, Germany; University of Tübingen, Germany

## Abstract

**Objectives:**

Obesity and its distribution pattern are important factors for the prediction of the onset of diabetes in humans. Since several mouse models are suitable to study the pathophysiology of type 2 diabetes the aim was to validate a novel computed tomograph model (Aloka-Hitachi LCT-200) for the quantification of visceral, subcutaneous, brown and intrahepatic fat depots in mice.

**Methods:**

Different lean and obese mouse models (C57BL/6, B6.V-Lep^ob^, NZO) were used to determine the most adequate scanning parameters for the detection of the different fat depots. The data were compared with those obtained after preparation and weighing the fat depots. Liver fat content was determined by biochemical analysis.

**Results:**

The correlations between weights of fat tissues on scale and weights determined by CT were significant for subcutaneous (r^2^ = 0.995), visceral (r^2^ = 0.990) and total white adipose tissue (r^2^ = 0.992). Moreover, scans in the abdominal region, between lumbar vertebrae L4 to L5 correlated with whole-body fat distribution allowing experimenters to reduce scanning time and animal exposure to radiation and anesthesia. Test-retest reliability and measurements conducted by different experimenters showed a high reproducibility in the obtained results. Intrahepatic fat content estimated by CT was linearly related to biochemical analysis (r^2^ = 0.915). Furthermore, brown fat mass correlated well with weighted brown fat depots (r^2^ = 0.952). In addition, short-term cold-expose (4°C, 4 hours) led to alterations in brown adipose tissue attributed to a reduction in triglyceride content that can be visualized as an increase in Hounsfield units by CT imaging.

**Conclusion:**

The 3D imaging of fat by CT provides reliable results in the quantification of total, visceral, subcutaneous, brown and intrahepatic fat in mice. This non-invasive method allows the conduction of longitudinal studies of obesity in mice and therefore enables experimenters to investigate the onset of complex diseases such as diabetes and obesity.

## Introduction

The modern life style has led to a high occurrence of obesity already reaching epidemic dimensions [1], [2]. Obesity, resulting from an imbalance between energy intake and expenditure, has often been associated with co-morbidities such as hypertension, sleep apnoe, dyslipidemia, and coronary heart disease, all together adding to the status of the metabolic syndrome [3], [4]. However, nearly one-third of obese individuals are considered as metabolically benign indicating that not the total amount of fat but the fat distribution determines the metabolic profile [5]. Subcutaneous fat storage is regarded as favourable and protective against impaired insulin sensitivity while an increase in visceral/intra-abdominal fat stores as well as ectopic fat storage in liver, skeletal muscle and pancreas is associated with an increased risk for the development of type 2 diabetes [6]. In addition, another parameter that showed a high association with the metabolic syndrome is the prevalence and intensity of non-alcoholic fatty liver disease [7]. Recently, an additional fat depot, the brown adipose tissue (BAT), raised the interest. It has been described mainly in small mammals, where it is responsible for non-shivering thermogenesis [8] via uncoupling of the respiratory chain [9]. However, new data indicated that BAT also exists and is functional in human adults [10], [11]. The amount of BAT is inversely correlated with the body mass index, suggesting a potential role of this fat depot in human energy metabolism [12]. Therefore, mechanisms to induce BAT development and activity are of major interest to dissipate excess energy as heat to prevent or ameliorate obesity [13], [14]. To asses individual risk parameters the non-invasive quantification of these storage sites is obligatory. The golden standard for the determination of abdominal adiposity and liver fat content in humans are MRI and CT [15].

Rodents, especially mice, are often utilized in research to investigate environmental and genetic impact under controlled conditions [16]. Body fat content in mice is commonly determined by dual energy X-ray absorptiometry (DEXA) and quantitative magnetic resonance (QMR) [17] but these technologies do not distinguish between fat depots. Although MRI and CT have generally been available in small animal research there are few studies describing the determination of body fat distribution in mice [18]–[21]. Therefore, the validation of methods already applied in humans is necessary to obtain data resembling the human situation. Moreover, there are no publications describing *in vivo* quantification of liver fat or BAT in mice by CT. In this study an advanced model of a rodent CT scanner, LaTheta LCT-200 (Hitachi-Aloka, Tokyo, Japan), was applied to identify and quantify subcutaneous (scWAT) and visceral white adipose tissue (vsWAT) and primarily liver fat and BAT in mice.

## Methods

### Animals

C57BL/6J (B6) mice were bred in our own facility based on founders from Jackson Laboratories (Bar Harbor, ME, USA). New Zealand Obese/HIBomDife (NZO, Nuthetal, Germany) mice originated from our own colony. B6.V-*Lep^ob^* (ob/ob) mice were also bred on site, using founders from Charles River (Sulzfeld, Germany).

Mice were housed in a controlled environment (20±2°C, 12 hr/12 hr light/dark cycle) and had free access to water and diet. Male and female animals at various ages were kept on different diets (standard diet - V153x R/M-H, Ssniff, Soest, Germany, high-fat diet - D12492, Research Diets, New Brunswick, NJ, USA and carbohydrate-free diet [22]) in order to obtain a wider range of body weights. Prior to CT scanning animals were anesthetized by inhalation of isoflurane (Forene®, Abbott, Wiesbaden, Germany) and maintained under isoflurane narcose during CT scan or sacrificed by cardiac puncture or cervical dislocation. Tissue collection was performed directly after scanning. The animals were kept according to the NIH guidelines for care and use of laboratory animals; all experiments were approved by the ethics committee of the State Agency of Environment, Health and Consumer Protection (State of Brandenburg, Germany).

### Computed tomography of mice

We validated a 3^rd^ generation computed tomography scanner, LaTheta LCT-200 (Hitachi-Aloka, Tokyo, Japan). The tube voltage was set at 50 kV and the current was constant at 0.5 mA. Animals were scanned in a 48 mm wide specimen holder with a resolution of 96 µm pixel. For all scans the same number of views (796) was used, which represents the number of data collected during a single 360° rotation around the object. According to the manufacturer the estimated radiation exposure of scanned objects remained below 40 mSv. During pilot experiments optimal scanning conditions were evaluated for each tissue and final conditions are presented in **Table 1**. Mean acquisition time for the scans of white adipose tissue was ∼13 minutes, interscapular brown adipose depot detection took approximately 15 minutes whereas the determination of liver fat content required a scanning time of 20 minutes.

**Table 1:** Scanning parameters for diverse fat depots.

Scan	Density range (HU)	Slice thickness (µm)	Slice pitch (µm]
**WAT**	(−500)–(−120)	192	600
**BAT**	(−120)–(0)	384	384
**Liver**	(−500)–(+350)	384	384

**HU** – Hounsfield Units, **WAT** - white adipose tissue, **BAT** – brown adipose tissue, **Liver** – liver, spleen and referent WAT fat depot.

Firstly, an overview scan of the whole mouse was created to allow the selection of regions of interest for the different scans. During all scans animals were placed on their back with face up and head front. Hind limbs were extended and fixed to specimen holder resulting in an angle of 90° between femur and spine (19).

Whole-body scans refer to the area between the proximal end of the first vertebra and the distal end of the tibia. Tail, feet and head were excluded as they only contain neglectable amounts of fat [20]. To quantify visceral and subcutaneous fat depots the area between the proximal end of lumbar vertebra L1 and the distal end of L6 was scanned. To detect the interscapular depot of brown adipose tissue the region ranging from the top of the shoulders to the proximal part of the liver was chosen. For liver fat quantification the areas that include liver and spleen (between the cranial part of the diaphragm and the lumbar vertebra L3) and adipose tissue (narrow area in the region of the lumbosacral joint) were scanned.

Abdominal muscle was used as a discriminant between visceral and subcutaneous fat depots [23]. Although, in general, the recognition of abdominal muscle is embedded in the LaTheta software, this automatic procedure needed numerous manual corrections. The commonly used fat density factor of 0.92 g/cm^3^ [24] is implemented in the software and used to calculate fat weights.

### Technical issues

Computed tomography recognizes different tissues upon their attenuation for x-rays, which is expressed in Hounsfield units (HU). The density between air (−1000 HU) and organs that are located adjacent to air and air pockets e.g. lung (−50 to +200 HU) exhibit a large difference in density. Therefore, the border area is partly misrecognized as mean value of both densities and might be detected as adipose tissue (−500 to −120 HU). The problem could partly be solved in skin and intestine by setting air boundary within 3 pixels from the pixels with attenuation of −700 HU or less. Furthermore, spinal cord and sternum cartilage were sometimes misrecognized as fat tissue, especially in lean mice. To rule out these sources of error a time consuming manual slice by slice correction was required especially around lung tissue.

### Comparison of simplified and detailed manual correction of whole-body scans

To reduce time needed for scan analysis we considered the whole lung as lean tissue and compared these results with detailed analyses in which we only corrected those as fat misrecognized parts of lung, and presented them as coefficients of variation (CVs) between both analyzing methods. For this purpose we used a subset of eight lean and normal weight (body weight: 24.6–28.5 g) and eight obese (body weight: 35.2–39.1 g) B6 mice.

### Comparison of the analysis of each slice versus every 3^rd^ slice for whole-body scans

Whole-body scans in mice under described conditions produce approx. 150 slices per mouse. Analysis and correction of all these slices exceeds 1.5 h per animal. We randomly chose 16 scan series of B6 mice and compared analysis of every single slice (slice thickness/pitch ratio: 192 µm/600 µm) with the analysis of every third slice of the same scans (corresponds to slice thickness/pitch ratio: 192 µm/1800 µm).

### Test-retest-reliability

In order to compare the novel LCT-200 with the previous model (LaTheta LCT-100) we conducted this test according to Hillebrand *et al.* [21]. B6 mice (n = 8, body weight: 24.6–38.8 g) were sacrificed and immediately afterwards the abdominal region between L1 and L5 was scanned for three times. The first two scans were done successively, without moving the mice. The third scan was done after the animals were repositioned and the region L1 to L5 was marked anew. Calculated CVs between 1^st^ and 2^nd^ scan represent consistency of measurements at two different time points. CV between 2^nd^ and 3^rd^ scan represent variation caused by different experimenters. The same test was done accordingly for BAT with eight B6 mice (body weight: 21.5–31.5 g), and for liver fat with eight NZO and B6 mice (body weight: 25.2–73.6 g).

### Inter-experimenter variability of analysis

Nine randomly chosen scan series of white adipose tissue, 27 scan series of BAT and 14 scan series for liver fat were analyzed, according to our protocol, by two or three different experimenters. The inter-experimenter variability was calculated as CV between results of analyses.

### Accuracy

Eleven mice (B6, ob/ob and NZO) of different body weights were scanned. Immediately afterwards fat pads were harvested and weighted (Sartorius VWR-153, Goettingen, Germany). Correlations were calculated between measurements on balance and weights of fat pads estimated by CT.

### Estimation of whole-body fat distribution from abdominal scans

Whole-body fat distribution can be estimated from short abdominal scans [25], [26]. This reduces scanning time and irradiation exposure of subjects. For verification we plotted the amounts of vsWAT and scWAT of two abdominal regions (L1–L6; L4–L5) against the results from whole-body scans.

### Liver fat

In human diagnostics CT scanners are already in use for the determination of liver fat content [27]. Due to relatively high deviations in tissue attenuations between different mice we normalized density values of liver against spleen and fat (regarded as fat free and absolute fat tissues, respectively), according to the following formula suggested by the manufacturer:
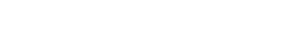
For the analysis we used mean HU values from 2 to 5 slices of central parts of each tissue as representative values.

In order to maximize the range of hepatic fat content in mice we chose several mice (n = 24, body weight: 26.3–68.5 g) of diverse backgrounds (B6, ob/ob and NZO) kept on different diets. Immediately after CT scan mouse livers were harvested and snap-frozen in liquid nitrogen. Approx. 30 mg of pestled liver was weighted on an analytical scale (AT-200, Mettler Toledo, Zurich, Switzerland) and homogenized in buffer (10 mM sodium dihydrogen phosphate, 1 mM EDTA, 1% polyoxyethylene(10)tridecyl ether). After centrifugation, triglycerides were determined by a commercial kit (Randox TR-210, Crumlin, UK). Laboratory results were plotted against values obtained by CT.

### Estimation of brown adipose tissue mass from interscapular scans

The weight of BAT from CT scans was assessed by applying the freely down-loadable image analysis software package ImageJ (National Institute of Health, USA) modified according to Dello *et al.* [28]. Briefly, CT images were imported into the software and the interscapular BAT depot was outlined manually on each slide. The obtained areas were summed up and multiplied by the slice thickness and the density of adipose tissue (0.92 g/cm^3^).

To test the accuracy of these scans BAT was dissected, separated from connective tissue and weighted on an analytical scale (AT-200, Mettler Toledo, Zurich, Switzerland). The values were correlated with BAT weights obtained by CT analysis.

Subsequently, the excised BAT was inserted into the abdominal cavity, below the liver to correlate the amount of BAT measured on scale with the CT estimate of dissected fat pad placed under the liver. To investigate whether BAT and WAT can accurately be distinguished by the CT scanner, the dissected BAT was repositioned into the lower abdominal cavity adjacent to the gonadal fat depot. Data analysis was performed as described above.

To analyse whether short-term cold exposure leads to a substantial alteration in the triglyceride content of BAT that can be visualized by CT measurement, a subset of B6 mice (n = 12, body weight: 21.5–31.5 g) were scanned before and after four hours of cold exposure (4°C). The mean HU value of the middle region of three central slices of BAT scans was determined and compared by repeated-measurement ANOVA.

### Validation of CT scans in living mice

Since the establishment of most adequate scan parameters and the validation of the scanner are time consuming processes we performed the experiments on sacrificed mice in order to minimize the exclusion of stressed animals related to repeated anaesthesia and irradiation. In order to verify differences in the quantification of fat depots between living and dead mice we used B6 mice and scanned interscapular (n = 8, body weight: 21.5–31.5 g ) and abdominal regions (n = 15, body weight: 17.8–32.7 g) under isoflurane anaesthesia. Subsequently, the animals were sacrificed and the scans were repeated in the same regions. Artefacts due to body motions in living animals were minimized by respiratory-gated scans and integration of four signal averages for BAT and two signal averages for WAT. Coefficients of variation were calculated between results of both analyses.

### Statistics

Linear regression analyses and repeated-measurement ANOVA were done using Sigma Plot 11.0 (Systat Software, San Jose, CA).

## Results

### Comparison of simplified and detailed manual correction of whole-body scans

For limiting the time required for an optimal determination of body fat distribution we compared results of simplified and detailed analyzing methods performed on obese and lean mice. Results are presented as coefficients of variation in **Table 2**. We used two obese models, the NZO as a model for polygenic obesity and the ob/ob mouse, carrying the leptin mutation on the C57BL/6 background, because both mice differ in their shape. The NZO for instance is much larger and has more lean mass than the ob/ob mouse. The values for the lean mice are slightly higher (<1.8%) than in the obese group (<1.3%), but generally there were no significant differences between both analyses.

**Table 2:** Coefficients of variance (CV) between simplified and detailed corrections of whole body scans.

	Fat ratio	Fat mass	Lean mass	Fat+lean mass
**Obese**	1.06±0.23	0.15±0.02	1.31±0.31	0.94±0.22
**Lean**	0.97±0.23	0.26±0.13	1.79±0.47	1.68±0.43

Eight C57BL/6 mice per group; CVs presented as mean (%) ± SEM.

### Comparison of the analysis of each slice versus every 3^rd^ slice for whole-body scans

In order to further minimize the time needed for scanning and data analysis we compared the results obtained from 16 total scan series with those in which every 3^rd^ slice was evaluated. Differences in the amounts of fat and lean mass obtained by these two procedures were calculated as CVs (mean (%) ± SEM) and resulted in the following values: Fat ratio: 0.80±0.18, total fat: 0.47±0.12, vsWAT: 0.53±0.09, scWAT: 0.75±0.20 and lean mass: 0.52±0.08.

### Test-retest reliability and inter-experimenter variability of data analysis

The results for test-retest reliability are presented in **Table 3**. CVs for white adipose tissue CT measurements in the area L1 to L5 without repositioning of the animals ranged between 0.8% and 2.1% for the investigated parameters. After repositioning and anew marking of the area L1 to L5 CVs were comparable to the results obtained prior to repositioning (1.4% to 2.2%). Accordingly, CV for brown adipose tissue without repositioning was 1.61% and after repositioning CV was 2.11%. Values for liver fat with and without repositioning were 4.27% and 4.50% respectively.

**Table 3:** Coefficients of variance (CV) for test-retest reliability.

	not repositioned	repositioned	inter-experimenter
**Total fat**	0.80±0.26	1.88±0.52	0.35±0.06
**Visceral fat**	1.18±0.25	2.17±0.63	0.82±0.17
**Subcutaneous fat**	2.09±0.55	1.41±1.05	0.31±0.07
**BAT**	1.61±0.44	2.11±0.39	2.05±0.43
**Liver fat**	4.27±1.02	4.50±1.29	2.56±0.37

not repositioned **- representing consistency of measurements**, repositioned **– representing variations by different experimenters**, inter-experimenter **- representing variability of analysis conducted independently by two or three experimenters; Eight C57BL/6 mice per group; Values are presented as mean (%) ± SEM.**

Variations in analyses of the determination of total fat mass as well as of visceral, subcutaneous, brown and liver fat conducted by two or three different experimenters ranged between 0.31% and 2.56% (**Table 3**). Accordingly, inter-experimenter variability of data analysis was lower than CVs for test-retest reliability, which represents consistency of measurement by this CT model.

### Accuracy of CT scans of white adipose tissue

**Figure 1:**
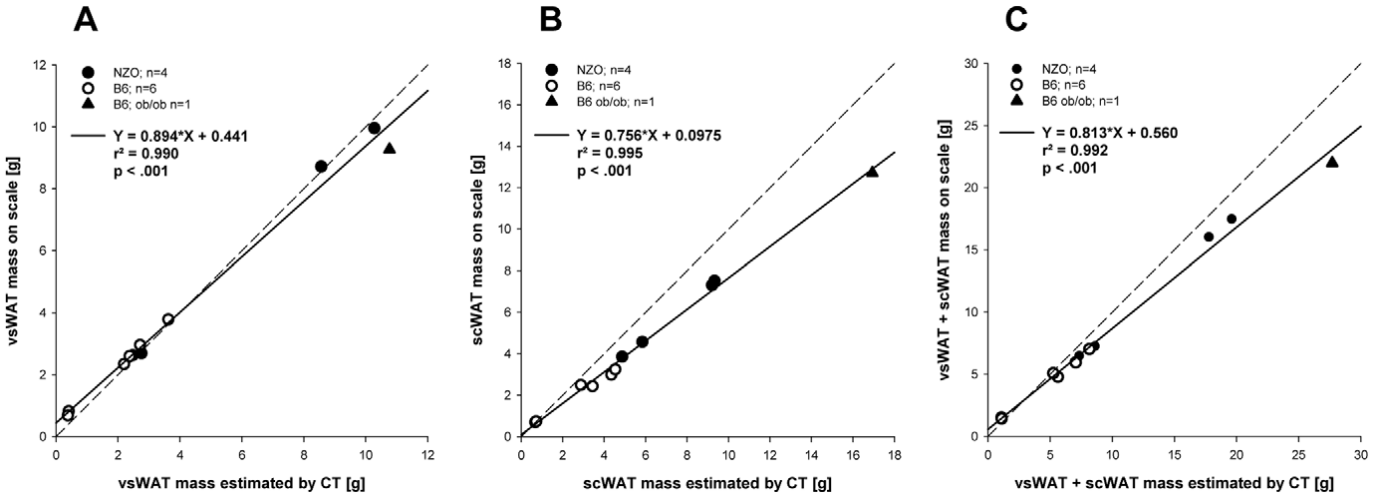
Resected white adipose tissue. Correlation between resected white adipose tissue weighted on scale and estimations of fat depot weights by CT. (A) Visceral adipose tissue (vsWAT), (B) subcutaneous adipose tissue (scWAT), (C) total fat (visceral and subcutaneous adipose tissue); r^2^ - coefficient of determination, dashed line – identity line.

*In situ* CT estimates were plotted against *ex vivo* weights of harvested fat pads. Correlations were calculated for visceral (vsWAT), subcutaneous (scWAT) and total fat mass (vsWAT+scWAT). Correlation between *in situ* and *ex vivo* fat masses was very high (vsWAT, scWAT, vsWAT+scWAT: all r^2^ = 0.99) (**Figure 1**). The recovery rate, measured as a proportion between the values estimated by CT and the fat mass measured on a scale were 1.18 g/g for vsWAT, 0.81 g/g for scWAT, and 0.95 g/g for total fat.

### Estimation of whole-body fat distribution from abdominal scans

**Figure 2:**
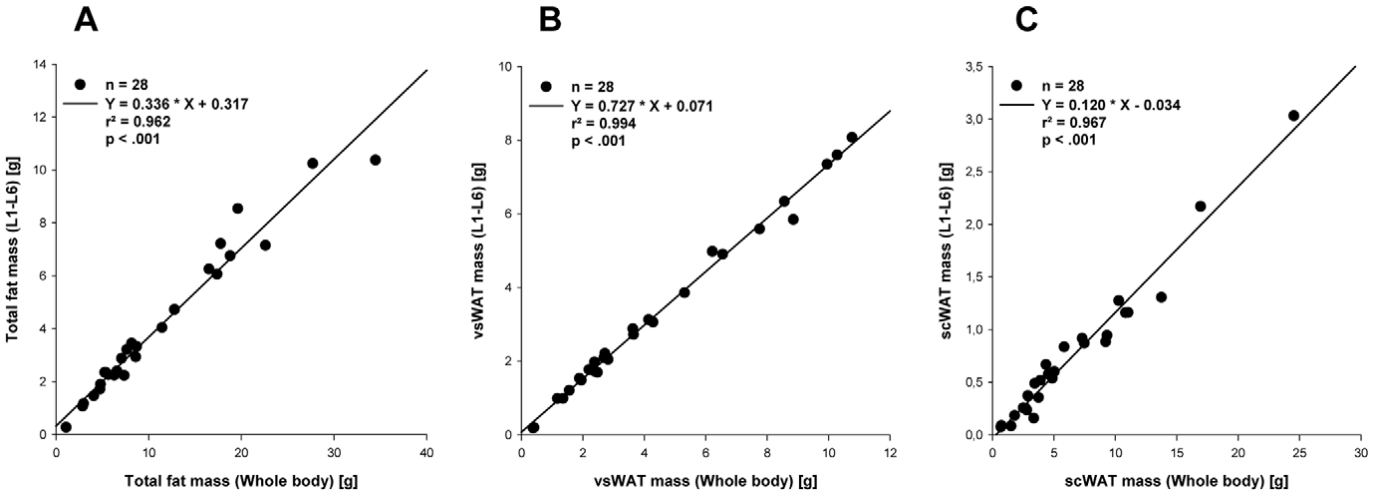
Abdominal scan (L1–L6). Correlation between weights of fat depots in whole body scans and in scans of abdominal area between lumbar vertebrae L1 to L6. (A) Total fat mass (vsWAT+scWAT), (B) visceral adipose tissue (vsWAT), (C) subcutaneous adipose tissue (scWAT); r^2^ - coefficient of determination.

Several mice (n = 28, body weight: 22.2–72.1 g) of different strains (B6, NZO, ob/ob) were scanned and the fat distribution of the whole-body was compared with the fat distribution in abdominal areas located between lumbar vertebrae L1 and L6 or L4 and L5. The results showed excellent correlations between the fat amount in the L1 to L6 region and in whole-body scans (r^2^_total fat_ = 0.962, r^2^_vsWAT_ = 0.994), presumably because most of vsWAT is located in this region. However, scWAT in the same region also correlated well with whole-body scans (r^2^_scWAT_ = 0.967) (**Figure 2**). Correlations between whole-body scans and L4 to L5 region were not as good as for L1 to L6 region but still acceptable (r^2^_fat ratio_ = 0.875, r^2^_total fat_ = 0.948, r^2^_vsWAT_ = 0.988, r^2^_scWAT_ = 0.904) (**Figure S1**). Consequently, total fat mass can be predicted by abdominal scans. Lean mass in the abdominal region did not correlate well with whole body values (r^2^_L1–L6_ = 0.103, r^2^_L4–L5_ = 0.605) and therefore, correlation coefficients for fat ratio were not as high as for fat mass (r^2^_L1–L6_ = 0.918, r^2^_L4–L5_ = 0.875).

### Determination of liver fat content by CT

**Figure 3:**
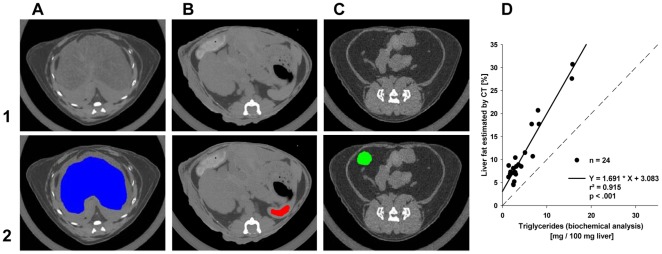
Quantification of hepatic fat by CT. Selected areas of liver (A; blue), spleen (B; red) and WAT (C; green) for determination of mean HU values. upper panel (1): raw gray scale scan slices, lower panel (2): selected organ parts used in calculation of liver fat. (D) Relationship between amounts of intrahepatic fat isolated and quantified with biochemical analysis and estimations by computed tomography. Dashed line – identity line, r^2^ - coefficient of determination.

In order to test whether liver fat content can be detected efficiently by the LaTheta LCT-200 we compared results obtained by CT scans with those determined by biochemical measurement of hepatic triglycerides. The correlation was high (r^2^ = 0.915). However, results obtained by CT exceeded results of biochemical triglyceride extraction as indicated by a regression slope of 1.7 (**Figure 3**).

### Quantification of the mass of brown adipose tissue by CT

**Figure 4:**
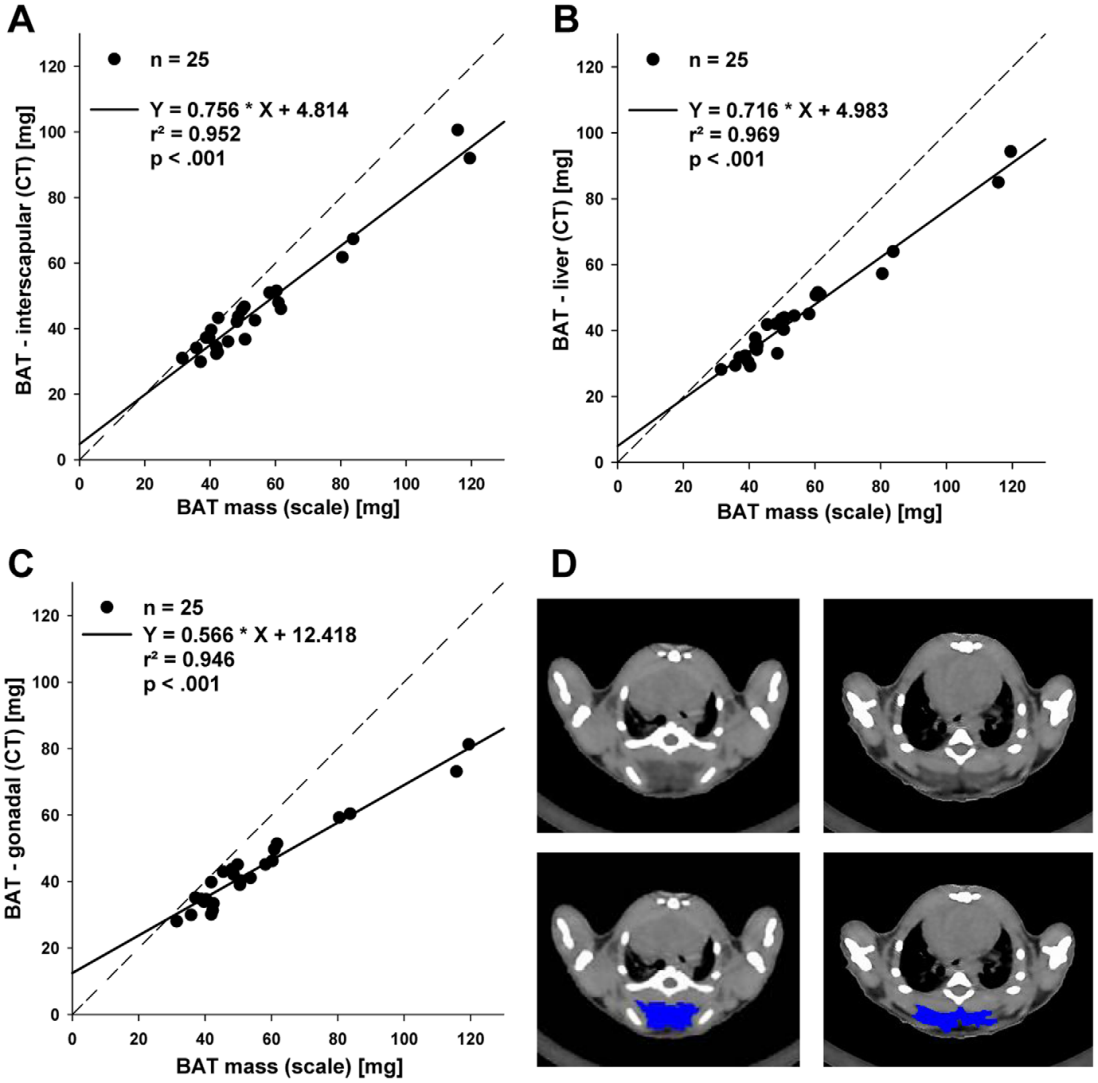
Brown adipose tissue. Correlation between resected brown adipose tissue (BAT) weighted on scale and estimations of fat depot weights by CT. (A) BAT depot in situ (interscapular), (B) resected BAT depot inserted under the liver, (C) resected BAT depot inserted in gonadal fat depot; dashed line – identity line, r^2^ - coefficient of determination. (D) Analysis examples of two different slices of interscapular brown adipose tissue depot by ImageJ (NIH) program. Upper panel: raw gray scale scan slices, lower panel: manually outlined and selected BAT in ImageJ (NIH).

To evaluate the quantification of interscapular BAT depot we scanned 25 mice of different body weights (11–36 g) in the region ranging from the top of the shoulders to the proximal part of the liver and compared the results with the balance weight of the corresponding BAT preparations. The results indicate a strong linear relation (r^2^ = 0.952). The high coefficient of determination for the amount of BAT repositioned below the liver (r^2^ = 0.969) or into the lower abdominal cavity (r^2^ = 0.946) and the resected BAT placed on the scale proved a high accuracy of detection (**Figure S2**). However, the CT scanner underestimated the amount of BAT as the slopes of the regression lines were below 1.0 (**Figure 4**). CT measurements of interscapular BAT before and after four hours of acute cold exposure resulted in a significant increase in mean HU values (before: −49.38±39.4; after: 16.13±12.31; p<0.001).

### Validation of CT scans in living mice

Body motion, especially in thoracal region caused by breathing and cardiac motion in mice could lead to disrupted scans. In order to test whether results differ between scans performed in living anesthetized and dead mice we studied fat distribution of 15 living mice and repeated the scans after sacrificing the mice. Results of the comparison of scans are presented as CVs between measurements. All values obtained (total fat: 1.41%, vsWAT: 1.93% , scWAT: 0.88%, BAT: 2.05%) are lower than CVs of test-retest reliability, demonstrating that CT is indeed a non-invasive method that can be used for longitudinal studies in order to follow changes in fat distribution.

## Discussion

The ability to monitor the development of obesity and liver steatosis and to determine the amount of brown adipose tissue in mice is crucial in obesity and diabetes research. Although CT imaging technique of fat depots is considered a golden standard in human diagnostics [15] the application of this method is still under development in laboratory animals. Here we present the validation of this non-invasive method in mice using the novel LaTheta LCT-200 CT scanner for small animals and demonstrate for the first time that it allows the quantification of intrahepatic fat as well as of the interscapular BAT depot. This will enable researchers to obtain consecutive datasets on fat distribution in single animals in a non-invasive manner.

To compare this novel model of CT scanner with the former model (LaTheta LCT-100) we partly repeated previously reported experiments as described by Hillebrand *et al.* [21] regarding accuracy, sensitivity and reliability of measurements on white adipose tissue. Comparing our data on accuracy with data obtained with LaTheta LCT-100 we observed better correlations between CT estimates and balance weight of fat pads as well as lower standard estimations of the mean regarding test-retest reliability.

Scanning conditions proposed by the manufacturer were modified to obtain more accurate quantification of adipose tissue depots. Optimal attenuation coefficients for WAT were set at −500 to −120 HU and −120 to 0 HU for BAT. The threshold of −120 HU between WAT and BAT enabled a clear differentiation between highly merged white and brown adipose tissue in the interscapular region. Besides the WAT, the CT also identified parts of lungs, cartilage, spinal cord and skin within the same density range. These artifacts in whole-body scans needed manual correction which highly prolongs the time of analysis. In human diagnostics scans of particular abdominal regions show good correlation to whole-body scans [29]. To reduce scanning and analyzing time, animals' exposure to radiation, as well as analyzing errors, we propose to substitute whole-body scans with scans of the abdominal region between lumbar vertebrae L1 and L6 because they correlate very well with whole-body fat distribution and contain fewer artifacts. Furthermore, the scans between L4 and L5 also correlate well with whole-body scans and could supply a rough estimation of body fat distribution. However, it is possible that the mobility of gut and other organs within the peritoneum could affect the reproducibility of short range scans in mice. A slice thickness of 192 µm and a slice pitch of 600 µm provide very accurate images of mice. But, considering the low CVs we conclude that for fast quantification of visceral and subcutaneous fat depots it is sufficient to evaluate every 3^rd^ slice, or resultant to increase slice pitch to 1800 µm and analyze each slice. Scanning of liver required higher precision because fat accumulation in the liver is not always homogenous [30]. Scans of BAT, due to its small size, also had to be precise. Thus we used 384 µm slice thickness with the same pitch for these scans.

Abdominal muscle, considered as the edge between scWAT and vsWAT [31], was not always accurately recognized by the software as a continuous line, but required manual corrections. Due to this correction and others mentioned above, analysis of scans could have depended on the experimenter or even be different by one experimenter at different time points. To avoid this bias we created clear guidelines for precise scan analysis and tested inter-experimenter variability. The obtained results showed that analysis performed according to the protocol did not differ significantly between experimenters and can therefore be regarded as reproducible.

Due to practical reasons validation was conducted in dead animals. But in order to prove that this technique is also eligible for in-vivo studies we tested it in living animals and compared these results with those obtained immediately after sacrifice. Since the CVs between both subsets of scans for total, visceral, subcutaneous and brown adipose tissue are lower than CVs of test-retest reliability for repeated measurements we conclude that there is no significant difference in results for the quantification of fat in anaesthetised and dead animals.

The accuracy of CT measurements is supported by very high correlation coefficients between measurements of fat pads on scale and by the scanner. However, the ratio between these values was quite different for vsWAT and scWAT. This leads to the conclusion that systemic errors occurred during tissue preparations. Overestimation of scWAT mass by CT could be caused by incomplete preparation of fat pads due to its strong connection to the skin and connective tissue. On the contrary, it is easier to dissect visceral than subcutaneous fat depot, but it is difficult to separate it completely from connective tissue. This could lead to higher amounts of vsWAT on the scale than in scans.

Liver fat content correlates well with insulin resistance in lipodystrophic mice [32]. and appears to be a good predictor of type 2 diabetes mellitus in humans [33]. Although the intrahepatic fat in rodents can be precisely quantified with magnetic resonance spectroscopy [34], this method is expensive and not widely available. Here we presented a simple, quick and reliable method for liver fat content determination by CT. For more precise quantitative assessment of intrahepatic fat we used visceral fat and spleen as internal fat and lean references, respectively. However, pathological alterations in the liver might result in parenchymal changes and subsequent differences in HU values which could falsify the obtained results [35]. Therefore, to investigate a pathological status additional validations are needed.

A high correlation coefficient between the biochemical extracted liver triglycerides and CT analysis represented good overall sensitivity of the method. However, a regression slope of 1.7 indicates an overestimation of intrahepatic fat content by CT.

Our data show that the LaTheta LCT-200 also allows the quantification of interscapular BAT which has not been validated for the previous CT model (LaTheta LCT-100) [21]. Time intensive manual outline of each slice is necessary as the HU range of BAT varies largely between individuals (data not shown). This is most likely due to the fact that HU-values of BAT change with its activity [36] and may also vary with fat content of BAT caused by white adipocytes in BAT or alterations in triglycerides storage in BAT cells. These incorporated white adipocytes, especially in obese mouse models, are the reason why the upper limit of weight range for the detection of BAT is restricted as both the tissue dissection as well as the visual distinction of grey shades becomes more and more difficult. The underestimation of the amount of BAT by CT could be caused by connective tissue attached to this adipose tissue depot which is difficult to visualize by CT but easier to dissect. The BAT mass was calculated using the density of WAT, but the density of BAT is probably higher than of white adipose tissue due to the lower total fat content and different size of fat droplets. In addition, the density of BAT might depend on acclimation status (amount of triglycerides stored) and its activity. To further analyse the sensitivity of BAT CT scans and to investigate the variation in triglyceride content of BAT, HU values before and after four hours of cold exposure were determined. Mean BAT CT radio density (in HU) increased substantially after cold exposure in B6 mice. This was already observed in human studies investigating the metabolism of BAT after cold exposure [37]. This shift of HU to higher values upon cold exposure is caused by the depletion of intracellular triglycerides stores in brown adipocytes which are used for an increased thermogenesis [38].

In summary, our data show that computed tomography is a valid method for quantification of total, visceral, subcutaneous and interscapular brown fat depots as well as of ectopic fat in the liver of lean and obese mice.

## Supporting Information

Figure S1 **Abdominal scan (L4–L5).** Relationship between weights of fat depots in whole body scans and in scans of abdominal area between lumbar vertebrae L4 to L5. (A) Total fat mass (vsWAT+scWAT), (B) visceral adipose tissue (vsWAT), (C) subcutaneous adipose tissue (scWAT); r^2^ - coefficient of determination.
(TIF)

Figure S2 **Scan of reinserted dissected interscapular brown adipose tissue.** (1) – under the liver, (2) – in gonadal fat depot; Upper panel: raw gray scale scan slices, lower panel: manually outlined and selected BAT in ImageJ (NIH).
(TIF)
