# Supplementary figures and images for: Non-Invasive Quantification of White and Brown Adipose Tissues and Liver Fat Content by Computed Tomography in Mice
Source: PLoS One. 2012 May 16;7(5):e37026. doi: 10.1371/journal.pone.0037026 (PMC3353985; doi:10.1371/journal.pone.0037026)

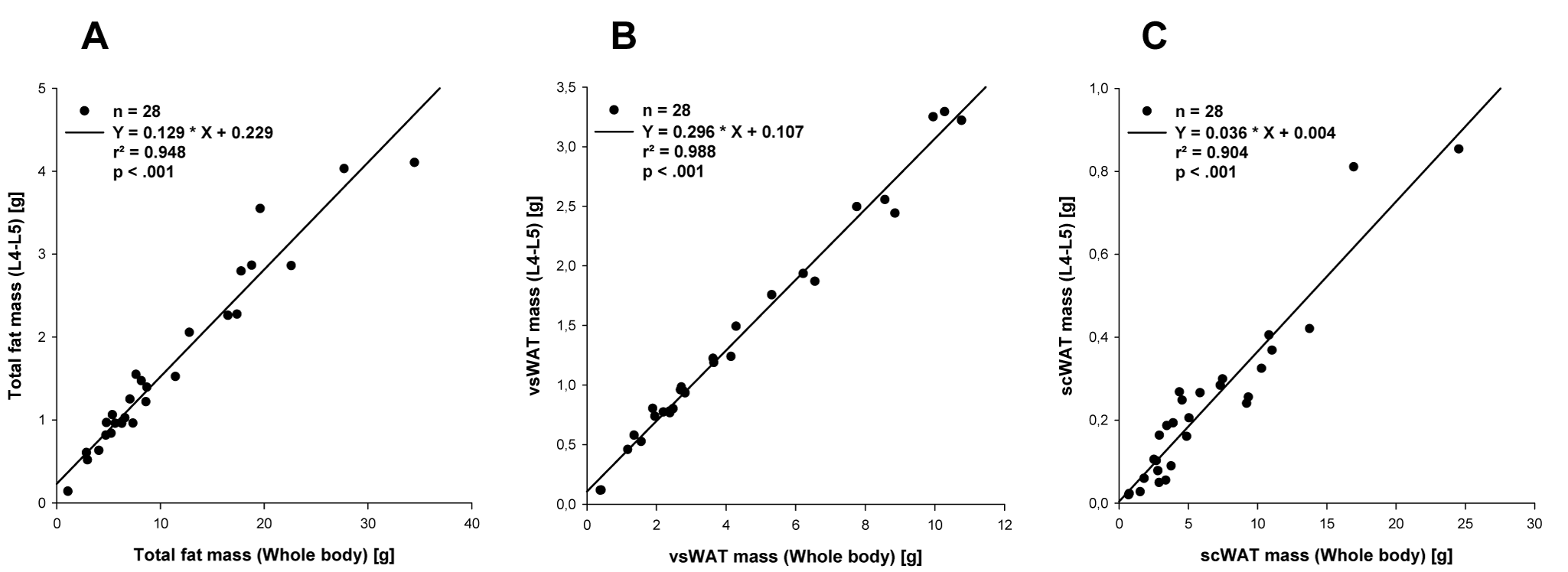

Supplement: Figure S1 — Abdominal scan (L4–L5). Relationship between weights of fat depots in whole body scans and in scans of abdominal area between lumbar vertebrae L4 to L5. (A) Total fat mass (vsWAT+scWAT), (B) visceral adipose tissue (vsWAT), (C) subcutaneous adipose tissue (scWAT); r2 - coefficient of determination. (TIF) [file pone.0037026.s001.tif]

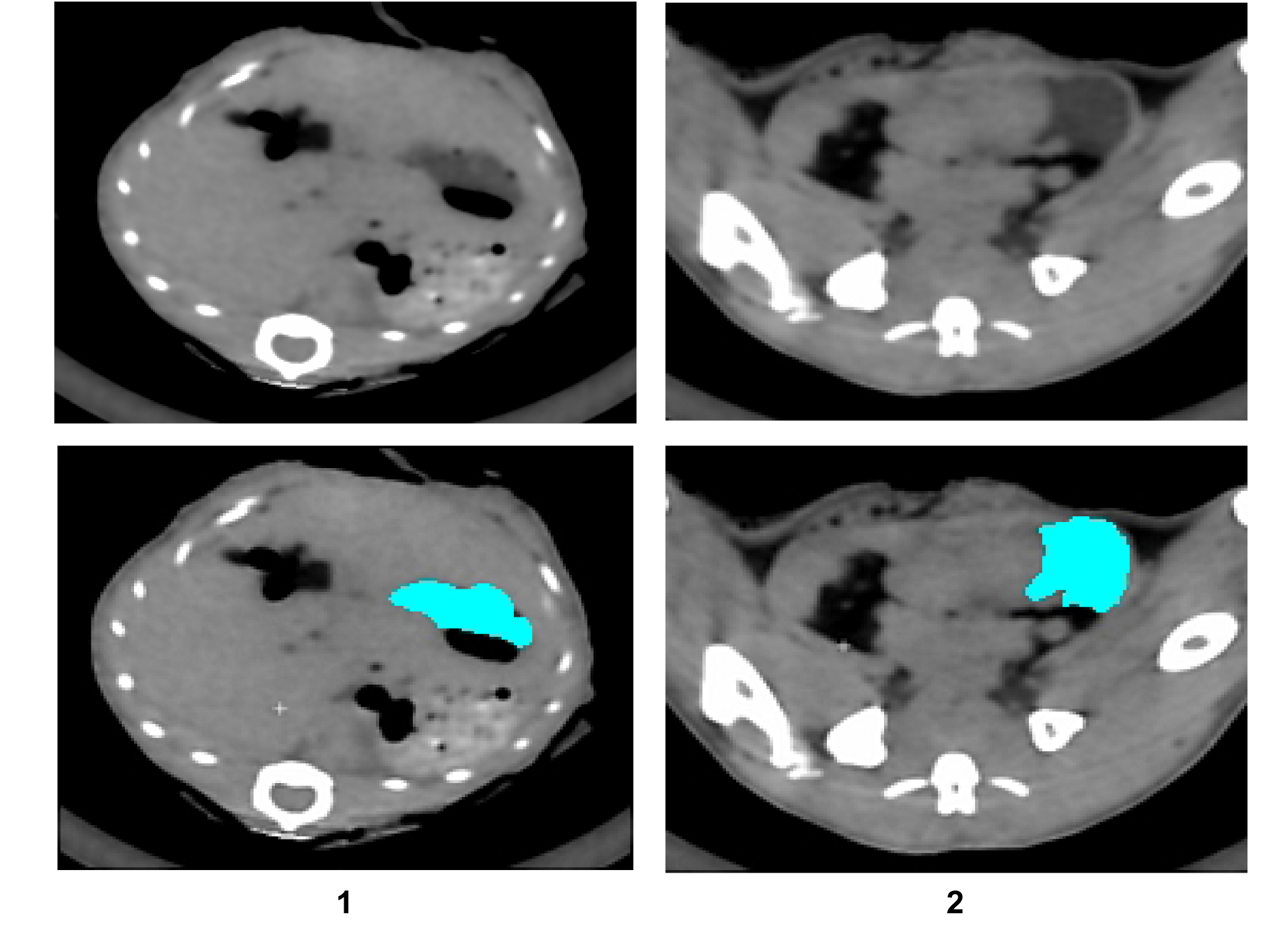

Supplement: Figure S2 — Scan of reinserted dissected interscapular brown adipose tissue. (1) – under the liver, (2) – in gonadal fat depot; Upper panel: raw gray scale scan slices, lower panel: manually outlined and selected BAT in ImageJ (NIH). (TIF) [file pone.0037026.s002.tif]
